# Sustainable biorefining in wastewater by engineered extreme alkaliphile *Bacillus marmarensis*

**DOI:** 10.1038/srep20224

**Published:** 2016-02-01

**Authors:** David G. Wernick, Sammy P. Pontrelli, Alexander W. Pollock, James C. Liao

**Affiliations:** 1 Department of Chemical and Biomolecular Engineering, University of California, Los Angeles, 5531 Boelter Hall, 420 Westwood Plaza, Los Angeles, CA 90095, USA; 2 Department of Chemistry and Biochemistry, University of California, 607 Charles E. Young Drive East, Los Angeles, CA 90095, USA; 3 UCLA-DOE Institute for Genomics and Proteomics, University of California, 201 Boyer Hall, 611 Charles E. Young Drive East, Los Angeles, CA 90095, USA; 4 Department of Bioengineering, University of California, 420 Westwood Plaza, 5121 Engineering V, Los Angeles, CA 90095, USA

## Abstract

Contamination susceptibility, water usage, and inability to utilize 5-carbon sugars and disaccharides are among the major obstacles in industrialization of sustainable biorefining. Extremophilic thermophiles and acidophiles are being researched to combat these problems, but organisms which answer all the above problems have yet to emerge. Here, we present engineering of the unexplored, extreme alkaliphile *Bacillus marmarensis* as a platform for new bioprocesses which meet all these challenges. With a newly developed transformation protocol and genetic tools, along with optimized RBSs and antisense RNA, we engineered *B. marmarensis* to produce ethanol at titers of 38 g/l and 65% yields from glucose in unsterilized media. Furthermore, ethanol titers and yields of 12 g/l and 50%, respectively, were produced from cellobiose and xylose in unsterilized seawater and algal-contaminated wastewater. As such, *B. marmarensis* presents a promising approach for the contamination-resistant biorefining of a wide range of carbohydrates in unsterilized, non-potable seawater.

Biorefining of cellulosic biomass is an important path towards sustainable production of fuels and chemicals. However, several issues remain a barrier to industrial biomass biorefining, amongst which are contamination risk, pentose and disaccharide co-fermentation, and water footprint^1^,^2^,^3^,^4^,^5^,^6^,^7^. Although cellulosic biomass stands as an abundant feedstock, synergistic conversion of its degradation products, cellobiose and xylose, is still a challenge^8^. Water use is also coming under scrutiny, as regions of the US and Brazil face record droughts^9^. The possibility of contamination further adds to the energy expenditure, time, and cost of biorefining processes^10^. Thermophiles have been proposed as contamination-resistant organisms, but their general sensitivity to oxygen and lack of genetic tools is slowing their development as biocatalysts^11^,^12^. Industrial lactic acid producers and the Brazilian ethanol industry use acidophiles for pH balance and contamination resistance^10^, but these strains have yet to show utility with complex substrates or wastewater streams instead of potable resources.

The ideal biocatalyst would be able to process cellulose degradation products in seawater or wastewater with low risk of contamination. As an extreme alkaliphile, *Bacillus marmarensis* shows promise as a contamination resistant bacterium. The multiple adaptations required to thrive in basic conditions are immensely rare, and no alkaline phages viable beyond pH 11 have been identified^13^,^14^. Additionally, *B. marmarensis* exhibits a rapid growth rate and is able to co-ferment the cellulose degradation products cellobiose and, contrary to literature reports^15^, xylose.

Use of *B. marmarensis* for industrial biorefining has not previously been explored. Here we have developed a set of genetic tools and a transformation protocol that will allow further engineering of *B. marmarensus* for specifically tailored applications. Using these genetic tools, and employing the unique properties of *B. marmarensus*, we have demonstrated the use of this strain for high-yield and high-titer production of ethanol within algal wastewater and seawater. This work serves to demonstrate *B. marmarensis* as an opportunistic host for next-generation bioprocessing.

## Results

### Alkaliphilicity

**Figure 1:**
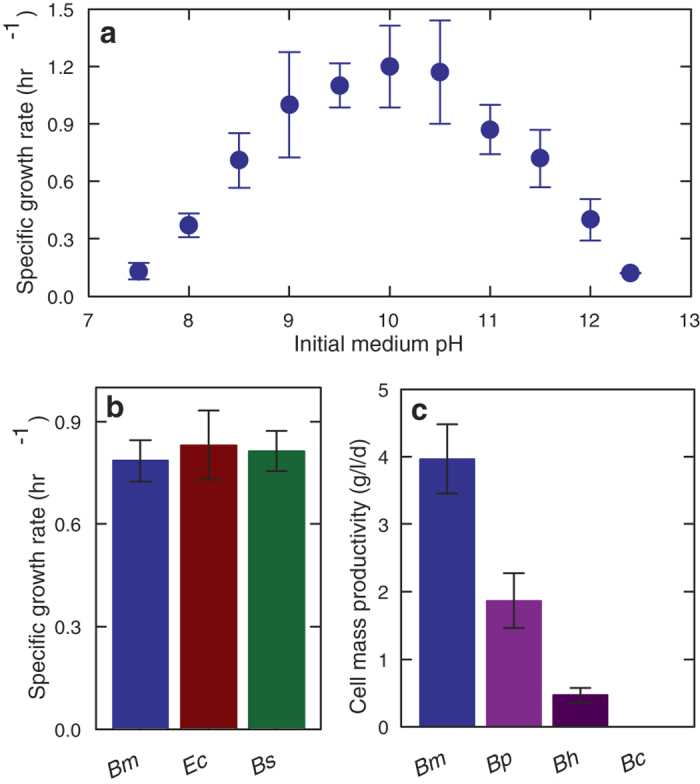
*B. marmarensis* thrives in alkaline conditions. (**a**) Specific growth rate of *B. marmarensis*; the maximum was observed between pH of 9.0 to 10.5. (**b**) Specific growth rate comparing *B. marmarensis* at pH 10 (Bm), to *Escherichia coli* (Ec), and *Bacillus subtilis* (Bs) at pH 7. No significant difference exists. (**c**) Cell mass productivity of extreme alkaliphiles at pH 11.4. Bp = *B. psuedofirmus* OF4. Bh = *B. halodurans* C-125. Bc = *B. clausii* KSM-K16. Bm accumulates cell mass more rapidly than the other alkaliphiles. N = 3 to 5. Error bars are S.D.

*B. marmarensis* attracted our attention from reports of its extreme alkaliphilicity; it demonstrated growth in medium with pH as high as 12.5^15^. To our knowledge, no strains able to grow in a higher pH medium have been identified. Previously, we sequenced and annotated its genome^16^. We began further study by characterizing its growth rate across a wide pH range (Fig. 1a). Following overnight acclimation at each pH*, B. marmarensis* grew most rapidly between pH 9.0 and 10.5. No growth occurred at pH 7.0 or beyond pH 12.5. Furthermore, exposure to neutral medium appeared lethal, as raising pH following neutral conditions did not render growth. The specific growth rate of *B. marmarensis* at pH 10.0 of 0.8 ± 0.1 hr^−1^ matched that of *Escherichia coli* and *Bacillus subtilis* in neutral conditions (Fig. 1b). Its cell mass accumulation greatly surpassed other known strong alkaliphiles, growing at twice the rate of model extreme alkaliphile *Bacillus pseudofirmus* (Fig. 1c).

### Contamination Resistance

With hearty growth under these conditions, we hypothesized that *B. marmarensis* should exhibit a level of contamination resistance. To test this, we propagated the strain in intentionally-contaminated medium and evaluated its prevalence. Two different contamination systems were established: one with open-air exposure as used to catch wild yeast^17^, and a second with moist, mountain soil directly added to the medium. *B. marmarensis* was inoculated into both systems at pH 9.5, pH 11.5, or pH 9.5 with daily sodium-carbonate washing, and re-propagated by daily 100-fold dilutions.

**Figure 2:**
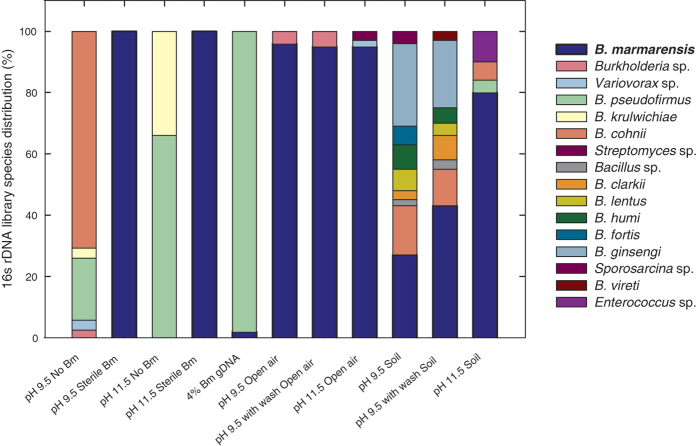
16s rDNA library of intentionally-contaminated *B. marmarensis* cultures demonstrates its resistance to competitors. A 1% inoculation of *B. marmarensis* was grown in media contaminated by outdoor exposure to air or addition of moist mountain soil. Cultures were diluted into fresh, unsterilized media daily for 7 days. Controls were performed in sterile media with and without *B. marmarensis*, and at both pH setpoints. A sensitivity control with 24:1 *Bh:Bm* genomic DNA (4% Bm gDNA) was also performed. The 16s rDNA library was quantified on final day. *B. marmarensis* dominates the open-air systems, and greater dominance with harsher alkaline conditions when exposed to soil. Together, this demonstrates *B. marmarensis’* potential to outgrow competitors in alkaline conditions.

After a week of growth, contamination was assessed by several visual and molecular evaluations. First, no fungal growth nor phage plaques were apparent (Supplementary Fig. 1). Also, fungus was not detected in quantitative-PCR analysis of internal transcribed spacer (ITS) ribosomal regions. Second, inspection for foreign bacterial colonies showed minute contaminates in open-air settings, and larger contaminates from soil cultures (Supplementary Fig. 1). To quantify contamination, we performed high-resolution melt analysis (HMRA) of 3 hypervariable 16s rDNA regions^18^. HMRA showed that open-air cultures drifted towards higher penetration of *B. marmarensis* over time in all pH conditions, with final melting points of each 3 regions matching its genomic DNA (Supplementary Fig. 2). Soil-contaminated cultures showed a range of foreign DNA, with pH 11.5 cultures showing the closest match to melting temperatures to that of *B. marmarensis* genomic DNA; these conditions appear to become enriched with *B. marmarensis* over time. We next built full 16s rDNA libraries from each culture (Fig. 2 and Supplementary Fig. 3). In all open-air systems, greater than or equal to 95% of reads belonged to *B. marmarensis*. The strain remained dominant with soil-contamination, but to a lesser extent; at pH 11.5, *B. marmarensis* represented 80% of the library reads. Remaining prevalent with this heavy soil contamination (2%w/v) presents a promising approach for the same resistance in biorefining.

### Genetic tools and fermentation conditions

With a characterization of this strain’s pronounced alkaliphilicity, we then proceeded to develop working genetic tools and carbon-efficient fermentation conditions to assess *B. marmarensis* as a chemical-production platform. Previously, alkaliphiles have been transformed with protocols dependent on a neutral pH step^19^,^20^,^21^. *B. marmarensis* is an obligate alkaliphile and cannot survive in neutral conditions. We therefore set about adapting transformation protocols to entirely alkaline conditions. Attempts to transform *B. marmarensis* using natural starvation^22^, auto-competency^23^, and heat-shock^24^ all failed to yield colonies. An alkaline electroproation protocol for gram-positive bacteria was performed, using glycine supplementation and addition of osmoprotectant D-sorbitol (adapted from ref. 20). However, two previously undisclosed problems complicate this procedure: *B. marmarensis* grows unpredictably in rich medium with osmoprotectants, and wash buffers with osmoprotectants rapidly acidify (Supplementary Figs 4 and 5). Pre-heating and pre-aerating the osmoprotectant rich media and inoculation of fresh colonies (<1 week old) gives reproducible and predictable growth of competent cells (Supplementary Fig. 5). Also, a pH 7.8 washer buffer with minimal addition of alkaline salts provides an optimal balance between *B. marmarensis* viability and electrical resistance. Additional optimization of the rescue media with 0.5 M sorbitol as the only osmoprotectant and a glycine treatment of 1%(w/v) for 50 min enables transformation efficiency of 1.0 × 10^5^ colony-forming units per μg DNA (Supplementary Fig. 5).

**Figure 3:**
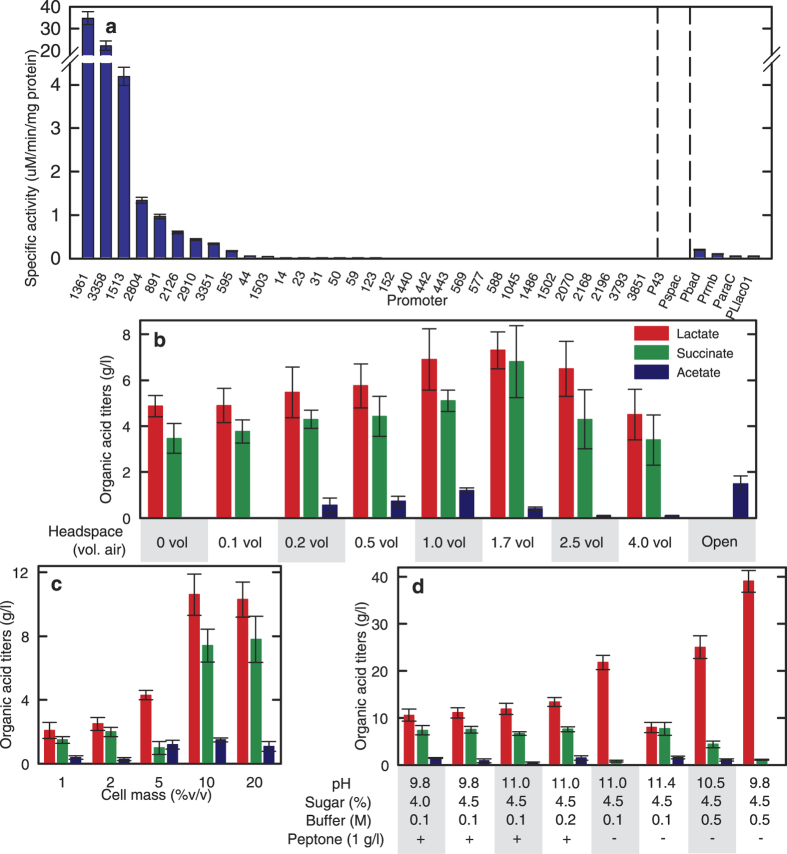
Genetic tool and fermentation condition discovery for use in alkaline biorefining with *B. marmarensis*. (**a**) Promoters were cloned from the genomes of *B. marmarensis*, *B. subtilis*, and *E. coli* and tested for their expression of native beta-galactosidase in *B. marmarensis. B. subtilis*/*E. coli* promoters are given by common names. *B. marmarensis* promoters are given annotation numbers (Further detail in Supplementary Table 1). (**b**) Fermentative production of lactate, succinate, and acetate from 4%w/v glucose in 48 hours. Providing 1.7 vol headspace air optimized organic acid production. (**c**) Increasing cell mass loading of cells greatly boosted organic acid yields. Productivity more than doubled-reaching maximum values at 14 hours instead of 48 hours. (**d**) Optimization of pH, buffer strength, and peptone concentration led to 94% conversion of glucose with high selectivity for lactate without acetate.

With a working transformation protocol, we then developed a library of promoters to drive genetic overexpression (Fig. 3a). A series of plasmids was constructed with different promoters driving expression of *B. marmarensis’* native beta-galactosidase (Supplementary Fig. 5). Promoters were selected from several genes predicted to express constitutively in *B. marmarensis* (Supplementary Table 1). Promoters were cloned as the approximately 400 base pairs upstream of the start codon to include both transcriptional elements and ribosome binding sites using beta-galactosidase as the reporter gene. Several promoters showed beta-galactosidase activity; promoters P_1361_ and P_3358_, regulating genes for a hypothetical S-layer protein and a glutamate dehydrogenase, respectively, provided the most activity (Fig. 3a). Interestingly, the strong promoters P_43_ and P_spac_ of *B. subtilis* were not active in *B. marmarensis*, while P_Llac01_ and other *E. coli* promoters showed some activity (Fig. 3a).

Utilization of these new metabolic-engineering tools requires carbon-efficient fermentation conditions, which may elude both aerobic and alkaliphilic bacteria such as *B. marmarensis*^25^. It has been shown that growth of alkaliphiles in extreme pH can give low cell mass yields^13^,^25^,^26^. This may be due to the thermodynamic challenge of generating a favorable proton gradient in alkaline conditions^27^,^28^. Cells require extracellular protons to pump across their membranes for ATP synthesis, but the very low concentration of protons in alkaline media compared to near-neutral cytoplasmic conditions makes this unfavorable. However, fermentative production is not reliant on growth^10^,^29^. In fact, de-coupling growth from fermentation could provide more carbon flux towards desired products instead of cell mass.

To evaluate the fermentative capacity of *B. marmarensis*, we quantified organic acids and alcohols produced in different growth media (Fig. 3b). The wild-type strain did not yield alcohols in any scenario. Cultures grown in rich tryptone and amino acid media yielded small quantities of lactate and succinate. In minimal media with glucose, *B. marmarensis* produced much greater quantities of lactate and succinate, in addition to trace amounts of acetate, depending on the headspace volume. Providing 1.7 vol air in the headspace of a sealed vessel without further aeration gave the highest titers with 7.3 ± 0.8 g/l lactate, 6.8 ± 1.6 g/l succinate and loading up to 10% v/v, at which it produced 10.6 ± 1.3, 7.4 ± 1.1, and 1.5 ± 0.1 g/l lactate, succinate, and acetate, respectively (Fig. 3c). Further optimization was performed from these conditions (Fig. 3d). It was found that increasing buffer strength, increasing sugar concentrations, adjusting pH, and removing peptone from the production media led to titers of 39.0 ± 2.3 g/l lactate and 1.1 ± 0.3 g/l succinate from 45 g/l glucose, and no measurable cell mass growth. This represents 90% conversion of carbon into fermentation products (comparable to 90–96% carbon conversions of the Brazilian ethanol industry, as described in ref. 10), demonstrates high selectivity for a single product, and provides a strong foundation for metabolic engineering of new bioprocesses featuring this strain.

### Engineering *B. marmarensis* for ethanol production in unsterilized, non-potable conditions

**Figure 4:**
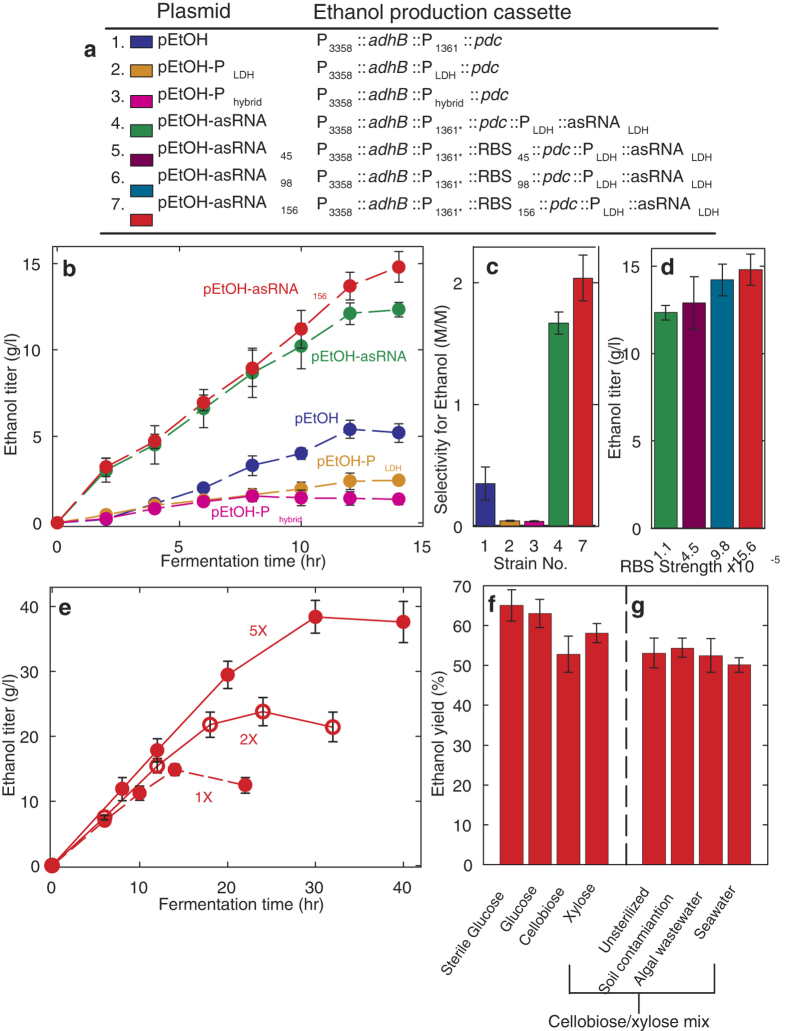
Engineering *B. marmarensis* for alkaline biorefining of complex carbohydrates in contaminated, wastewater medium. (**a**) Strain genotypes for alkaline ethanol production. All plasmids used *cat* selection marker and *repB* origin from ref. 4. P_hybrid_ was a fusion of P_1361_ and P_LDH_. P_1361’_ and P_ldh’_ had modified RBSs compared to their genomic counterparts. (**b**) Ethanol production of *B. marmarensis* strains from sterile glucose. Initial production with pEtOH reached 5 g/l and a 22% yield. Titers were bolstered with antisense RNA knockdown of lactate dehydrogenase (asRNA_LDH_). (**c**) Selectivity for ethanol over lactate in engineered strains. Increasing ethanol titers coincided with less lactate. (**d**) Raising RBS strength of *pdc* increased flux of pyruvate towards ethanol. Production rose to 15 g/l, or 65% the theoretical maximum. (**e**) Ethanol titers were raised to 38 g/l by concentrating glucose and salts in the medium. (**f**) Engineered *B. marmarensis* converted unsterilized cellobiose and xylose with similar yields. (**g**) Production ethanol from cellobiose and xylose co-fermentation with soil contamination and in algal wastewater or seawater.

To further demonstrate the utility of *B. marmarensis*, we engineered a strain to produce ethanol. We heterologously expressed pyruvate decarboxylase *pdc* and alcohol dehydrogenase *adhB* from *Zymomonas mobilis*^30^ to convert pyruvate to the in-demand biofuel ethanol. Ethanol production cassettes were constructed as given in Fig. 4a. Initial plasmid constructs expressed *adhB* by P_3358_ and *pdc* by P_1361_. *B. marmarensis* cells harboring this plasmid produced an ethanol titer of 5.2 ± 0.5 g/l in 14 hr, representing 22% of the theoretical maximum yield (Fig. 4b). High lactate titers made by this strain resulted in a low ethanol selectivity and yield. (Fig. 4c).

We attempted to increase ethanol yields by outcompeting lactate dehydrogenase (LDH) for pyruvate flux. We first aimed to increase *pdc* expression by finding a stronger promoter. Substituting P_1361_ for the native lactate dehydrogenase promoter (P_LDH_) decreased ethanol yields (Fig. 4b,c). Generation of a hybrid promoter splicing P_1361_ and P_LDH_ into P_hybrid_ also failed to increase yields. We therefore sought to disrupt the *ldh* gene encoding a native lactate dehydrogenase (LDH) using site-specific recombination^31^,^32^. A plasmid was constructed containing an ethanol production cassette flanked with a 500 bp region that is homologous to the *ldh* gene with the start codon removed. Transformation of this plasmid, followed by 3 serial dilutions and subsequent PCR based screening yielded a strain in which the *ldh* gene was disrupted with concatenated integration of the ethanol production cassette and selection marker. This strain demonstrated an 80% reduction in lactate titers, but production of ethanol was virtually eliminated. We therefore designed an antisense-RNA knockdown scheme^33^ (Supplementary Fig. 7). Incorporating this into the ethanol production cassette boosted titers to 12.3 ± 0.4 g/l, and greatly improved selectivity for ethanol over lactate (Fig. 4b,c). Furthermore, production titers were again raised by increasing the predicted ribosome-binding site translation-initiation rate (RBS, TIR). The RBS paired with P_1361_ was swapped for synthetic RBSs. Raising RBS TIR increased ethanol titers, with the strongest RBS increasing ethanol titers to 14.8 ± 0.9 g/l, or 65% the theoretical maximum yield (Fig. 4a,d). Through increasing the concentrations of glucose, salts, and metals, titers were raised to 38 ± 3 g/l ethanol (Fig. 4e). To mimic the Brazilian ethanol production process, cells were recycled by centrifugal separation from growth medium followed by a basic wash. At least 82% of the initial yield was produced in succeeding batches for up to 3 batches, presenting the opportunity to recycle *B. marmarensis* cell mass for multiple production rounds (Supplementary Fig. 8).

Yet to move towards a practical process, energy cannot be spent in sterilization and we must move away from glucose as the feedstock^34^. We repeated ethanol production from glucose medium devoid of decontamination. Previous experiments had been carried out without sterilization. Notably, ethanol yields remained within the established confidence intervals (Fig. 4f).

We replaced glucose with cellobiose and xylose. Both of these saccharides are major products of cellulose degradation. Their simultaneous conversion remains a challenge to biofuel production^35^. However, their consumption in a bioprocess may enable use of inexpensive cellulosic material as the feed, in place of more expensive glucose sources. *B. marmarensis* was previously shown to utilize cellobiose, but not xylose^15^. However, we predicted otherwise as we identified xylose isomerase and xylulose kinase genes within its genome^16^ that may allow it to assimilate xylose into the pentose phosphate pathway and central metabolism. During fermentations, *B. marmarensis* achieved yields of 53 and 58% the theoretical maximum from cellobiose and xylose, respectively, and 53% yield at a titer of 12.3 ± 1.2 g/l from a 50:50 mass mixture of both (Fig. 4f,g). No components in these fermentations were decontaminated, thereby, these yields were achieved from advanced feedstocks in unsterile medium.

Despite this success, such demonstration was not performed with a practical source of water. Drought conditions in much of the US necessitate use of wastewater or seawater – drinking water cannot be spared to make new energy sources. Additionally, no contaminates were intentionally introduced into the medium. We therefore continued testing cellobiose and xylose fermentations with intentional contamination and with wastewater (Fig. 4g). Adding soil to the fermentation had no significant effect as ethanol yields reached 54%. Next, algal wastewater replaced tap water in the medium, and no drop in titer was found with yields around 53%. Finally, seawater from the California coast was used as the water source. This marginally impacted production, with ethanol yields slightly falling to 50% from cellobiose and xylose in unsterilized conditions.

## Discussion

*Bacillus marmarensis* is an extreme, obligate alkaliphile that grows from pH 7.5 to 12.5, but cannot survive in neutral conditions. Its growth rate near the optimum medium pH of 10.0, is similar to that of *Escherichia coli* and *Bacillus subtilis* at pH 7 in rich medium. Its growth in rich medium at pH 11.4 greatly outpaces those of other alkaliphilic strains. This rapid replication maintained contamination resistance in intentionally adulterated medium.

With this remarkable finding, we developed a reliable electrotransformation protocol and a series of tools for genetic overexpression. Along with optimized fermentation medium and conditions, these tools enabled engineering of *B. marmarensis* for high yield (65% of the theoretical maximum) and high titer (38 g/l) ethanol production from glucose.

To initially assess industrial relevance of the strain, ethanol production from other carbon sources and without sterilization was performed. Medium intentionally contaminated with different natural samples did not affect product yields or titers. Furthermore, yields of 50% of the theoretical maximum or higher were obtained from intentionally contaminated xylose and cellobiose mixtures, suggesting further engineering may drive the strain for cellulose conversion.

Through these findings, we have shown the utility of *B. marmarensis* as a platform for alkaline biorefining. In prior work, the model gram-positive organism *B. subtilis* was engineered to produce 8.9 g/l ethanol from glucose at 89% of the theoretical maximum yield after 48 hr in carefully sterilized medium^36^. Although our yield did not quite reach 89%, our titer (38 g/l vs. 8.9 g/l) and productivity (1.25 g/l/hr vs. 0.19 g/l/hr) are several-fold higher with intentional contaminants. Scale-up and further optimization studies should improve all of these values towards profitable points such as those of the Brazilian ethanol industry^10^.

Further evaluation of this process suggests that, although the pH increase is drastically different than currently-employed processes (e.g. *Corynebacterium glutamicum* amino acid production at neutral pH, *E. coli* recombinant production at neutral pH^37^,^38^ and lactate production in acidic pH^39^), it does not introduce novel process complications. The cells cannot survive in neutral conditions in accidental release into nature, and bioreactor spills can be contained through medium neutralization. Medium neutralization is a risk control method already established for industrial catalytic reactions^40^. Additionally, although organisms that are as alkaline tolerant as *B. marmarensis* may exist naturally, they would not be abundant in carbohydrate or cellulosic feedstocks, as they tend to be found in alkaline and/or soda lakes^13^. It’s also improbable for a neutrophilic microbe to evolve alkaline tolerance, as multiple synergistic mutations are required. Alkaliphiles evolved overtime to code for alkaline-stable extracellular and membrane proteins (typically featuring greater arginine and lysine content), rapid Na^+^/H^+^ cycling through redundant antiporters, and specialized ATP synthases to survive in the inverse pH gradient (extracellular pH > intracellular pH)^13^,^19^,^20^,^21^,^25^,^26^,^27^,^28^,^41^. With these industrial considerations, and the engineering groundwork presented in this paper, *B. marmarensis* may have a promising future as a bioprocess host organism. It may become exceptionally beneficial for production of the alkaline amino acid products lysine and arginine, however further elucidation of the *B. marmarensis* amino acid metabolism must be performed before metabolic engineering these target compounds.

## Methods

### Strains, chemicals, reagents and plasmids

All chemicals and reagents were purchased from Sigma-Aldrich unless otherwise noted. *Bacillus marmarensis* DSM 21297 was obtained from DSMZ (Braunschweig, Germany). *B. pseudofirmus* OF4, *B. clausii* KSM-K16, and *B. halodurans* C-125 were obtained from ATCC (Manassas, VA – ATCC BAA-2126, 31084 and BAA-125, respectively). *Escherichia coli* strain JCL16 was used as described elsewhere^16^. *B. subtilis* 168 was obtained from BGSC (Columbus, OH, Strain 1A1).

All PCRs were first performed with Phusion DNA Polymerase (Fisher Scientific). If these reactions failed, PCRs were repeated with KOD Xtreme Hot-Start DNA polyermase (EMD Millipore). All primers where purchased through Integrated DNA Technologies (idtdna.com). Plasmids were assembled using T4 DNA polyermase (New England Biolabs) as follows: Plasmid DNA pieces were amplified by PCR to contain 15–30 base pair overhangs to match their neighboring fragments. Fragments were combined with splicing-by-overlap extension PCR (SOE-PCR) to yield 2–3 fragments per plasmids. Fragments were added in equimolar amounts to a T4 DNA polymerase assembly reaction with NEB Buffer #2 and 0.3 ul/10ul T4 DNA polymerase. Reaction volume was dependent on DNA concentration (in the range of 10–30 μl), as a minimum of 300 ng of each fragment was added. Reaction was incubated at room temperature for 5 to 10 mins. Plasmid assembly reactions were then transformed into *E. coli* XL1Blue (Agilent), selected on LB chloramphenicol at 20 ug/ml, and verified by colony PCR and Sanger Sequencing (Genewiz, San Diego, CA).

Ethanol production plasmids were cloned into pNW33N, as described above, with cassettes described in the text and using primers given in Supplementary Table 2. RBS sequences were identified on De Novo DNA (www.denovodna.com, ref. 42). The RBS of wild-type P_1361_ was found to be 5′-ACCATATTGGAGGTTATCTT-3′. The modified RBSs are as follows, with same labels as given in the text: RBS_156_ 5′-AATTTTAATAACACAGAAGAAGGAGGTAGAAA-3′, RBS_98_ 5′-CGTAAGTAAGAAAACACGAAGGAGGGAAGTT-3′, and RBS_45_ 5′-AAAAAGAAAACAAAGAAGGAGGGTAAAT-3′. For antisense knockdown of LDH, the LDH promoter was cloned to drive expression of the reverse complement of the LDH gene RBSs and first 20 or so codons (The *B. marmarensis ldh* gene was found to have 3 RBSs sequences upstream. This is given in Supplementary Fig. 7.

### Media, and growth rate and cell mass determination

All alkaliphilic strains were routinely cultured in alkaline LB broth (ALB) consisting of 10 g/l NaCl, 5 g/l yeast extract, 10 g/l tryptone, 4.2 g/l NaHCO_3_, and 5.3 g/l Na_2_CO_3_, with the exception of *B. clausii*, where NaCl, yeast extract, and tryptone were replaced with 8 g/l nutrient broth (all from BD Biosciences, NaCl from Sigma-Aldrich) and pH was lowered to 9.0. For plates, 15 g/l bacto agar was added (BD Biosciences). 5 μg/ml chloramphenicol was added when needed. For growth rate determination of *B. marmarensis*, a fresh plate was streaked from a freezer stock and grown overnight at 37 °C. Next morning, a single colony was inoculated into ALB broth and grown overnight at 37 °C and 250 rpm. Next day, a 1% inoculation was made into nutrient broth at each pH to be tested. Next day, cultures were diluted 100-fold and grown for 4 hours. Cultures were then diluted to same starting OD and growth was monitored overtime. Specific growth rate was calculated from each growth curve. For *B. subtilis* and *E. coli*, the same procedure was followed but all media was LB at pH 7.0. For cell mass determination, same procedure was followed for each strain, but cells were inoculated into 4% trypticase peptone at pH 11.4 and were grown overnight. Cell mass was harvested, washed with 1M NaHCO_3_ and then with MQ water, dried at 70 °C overnight, and quantified the next day. N = 5, error bars are standard deviation.

### Contamination studies

Flasks used in open-air cultures were left open and exposed near the intersection of 17^th^ St. and Montana Ave. in Santa Monica, CA. Here, a marine cloud layer formed each night as air swept in from the Pacific Ocean. Soil was obtained from a moist spot at the base of several plants in the Santa Monica Mountains (Malibu, CA). Soil was added at 2% w/v into media.

Media was ALB at pH 9.5 or 11.5. *B. marmarensis* was grown aseptically overnight in ALB, inoculated into unsterilized media in both systems, and diluted 100-fold with fresh unsterile media daily for one 1 week. For Na_2_CO_3_ wash, cells were harvested by centrifugation, suspended in 1M Na_2_CO_3_, incubated at room temperature for 20 mins, harvested again and diluted in fresh media. DNA was isolated from 1 ml daily samples using Qiagen QIAmp UCP Pathogen Mini Kit (#50214) with Qiagen Pathogen lysis tubes (#19092). All DNA extractions were normalized to a concentration of 1 μg/ml.

Plates were made with ALB at each pH. On the last day, an inoculation loop was immersed in the media and streaked across 3 plates. Plates were grown at 30 °C for 50 hrs before analysis. Following analysis, plates were grown another 72 hrs at 30 °C to further test for fungal growth. Fungal 18s rDNA intergenic sites were assayed with universal primer pair 5′-GGRAAACTCACCAGGTCCAG-3′ and 5′-GSWCTATCCCCAKCACGA-3′^18^, in Quantitative PCR described below. High-resolution melt analysis was an adaptation of the method of Cheng *et al.*^18^ modified to obtain melt curves as described below.

### Quantitative PCR and Melting-Temperature Determination

qPCR and melt temperatures were obtained with a Bio-Rad C1000 Thermal Cycler equipped with a CFX96 Real-Time System. All reactions were performed with Bio-Rad SsoFast EvaGreen Supermix. The reactions were 10 μl total consisting of 5 μl EvaGreen Supermix, 1 ng genomic DNA extractions, and 400nm primer pairs. Reactions were performed as follows: enzyme activation at 98 °C for 2 min, 40 amplification cycles of 98 °C for 5 sec and 60 °C for 5 sec, and a melt curve from 70.0 to 95.0 °C incrementing in 0.2 °C steps at 5 sec/step. All data was analyzed on Bio-Rad CFX Manager 2.0. For melt curves, N = 8 to 10. Fungal quantification was compared to a no-template control.

### 16s rDNA Library Assembly

DNA extractions were obtained from contamination studies (above). Samples were amplified using primers 5′-TCCTACGGGAGGCAGCAGT-3′ and 5′-GGACTACCAGGGTATCTAATCCTGTT-3′ from Brands *et al.*^43^ with KOD Xtreme Hot-Start DNA polymerase. Products were gel-electrophoresis purified on a 2.0% agarose gel run at 100 V in 0.5X TAE buffer for 30min. Bands were cut out and digested using Zymo DNA Clean & Concentrator kits. Bands were assembled into plasmids using Zero Blunt TOPO PCR Cloning Kit (Life Technologies, #K2800-20) and transformed into One Shot TOP10 Chemically-Competent *E. coli* cells (Life Technologies). Colonies were selected on LB Agar plates with 100 μg/ml ampicillin, 0.1 mM IPTG (Gold Biotech), and 0.1 mM X-Gal (Sigma-Aldrich). Plates were sent to Genewiz for sequencing of white colonies. Sequencing was performed on colonies using primer M13R 5′-CAGGAAACAGCTATGAC-3′. Sequencing reads with less than 500 base pairs were discarded. The library consisted of the first 100 high-quality reads generated for each sample. Species were identified with NCBI BLAST.

### Genetic transformation

*B. marmarensis* was streaked on an alkaline LB plate from a −80 °C freezer stock and incubated overnight at 37 °C. Single colonies were used to inoculate 2.5 mL culture in alkaline LB supplemented with 100 μg/mL kanamycin sulfate. That culture, and a separate sterile 250 mL shake flask containing 55 mL alkaline LB supplemented with 0.5 M D-sorbitol (wrapped with parafilm at the top) were incubated overnight at 37 °C.

The following morning, 750 μL of the 2.5 mL culture were used to inoculate the 55 mL shake flask (which evaporated to approx. 50 mL). The shake flask was incubated at 37 °C for 2.5 to 4 hr to reach an OD_600nm_ of 0.4 to 0.8. During this incubation, fresh wash buffer of 10% glycerol, 0.5 M D-sorbitol and 0.5 M D-mannitol (Sigma-Aldrich) was prepared. The wash buffer pH was raised to 7.8 with 0.3 N NaOH; pH was not allowed to rise above 7.8 during preparation to avoid excess salt accumulation. Once filter sterilized, the solution was kept on ice. Following the incubation, 5 mL of 10% glycine (pH 8.5–8.7) was added to the flask and incubated at 37 °C for 55 min. The flask was then placed on ice for 12–15 mins with periodic swirling to ensuring even cooling. Cells were kept on ice or in a chilled centrifuge through the remaining steps before incubation at 37 °C. 2 mL aliquots of the cells were then removed and spun down at 5,000 × *g* for 5 min at 4 °C. Cells were suspended in 1 mL wash buffer, and the wash was repeated for a total of 4 times. Finally, cells were suspended in 200 μL wash buffer.

Before transformation of plasmids, electrical resistance of the cells was tested and adjusted. 45 μL of cells were added to a 1 mm-gap electroporation cuvette which had been chilled on ice. Cells were pulsed with a Bio-Rad Micropulser at 2.7 kV. If the resulting time constant fell between 4.3 and 4.8 ms, the cells were acceptable. If the time constant fell below 4.3 ms, fresh wash buffer was added to the remainder of the cells and the change in time constant was measured. Once adjusted into the acceptable range, 5–50 ng of plasmid was added to 45 μL cells. Cells were transferred to a new, chilled 1mm electroporation cuvette. A single square-wave electric pulse was supplied by a Bio-Rad Gene Pulser Xcell system at 2.1 kV and 1.5 ms.

Immediately following the pulse, cells were rescued with addition of 250 ul room-temperature alkaline LB supplemented with 0.5 M D-sorbitol. Cells were incubated at 37 °C for 3–4 hr. 200–300 μL cells were plated on alkaline LB supplemented with 5 μg/mL chloramphenicol.

### Promoter and enzyme activity assays

Promoters were chosen from the *B. marmarensis* genome. They are given in Supplementary Table 1 with primers used for cloning and sequencing. The promoter was taken to be the 300–500 base pairs upstream of the start codon of their respective genes, and included the RBS. They were assembled into pNW33N plasmids (from ref. 4) to drive expression of beta-galactosidase from *B. marmarensis*.

Plasmids were transformed into *B. marmarensis*. Single colonies were inoculated in 3 ml ALB with chloramphenicol for growth overnight at 37 °C. Next morning, a 1% inoculation of the overnight culture was made into 50 ml fresh ALB media with chloramphenicol in a 250 ml baffled shake flask and grown at 37 °C/250 rpm until OD_600nm_ reached 1.0. Cells were spun down and suspended in 1 ml 50 mM Tris-HCl pH 7.5. Cells were lysed by Qiagen TissueLyser II by placing the cell suspension and 600 μl 0.1mM glass beads in a 2 ml screw top tube. Tube holders were pre-chilled at −20 °C for 20 mins. Cells were lysed for 5 mins at 30 Hz, chilled on ice for 5 mins, and again lysed for 5 mins at 30 Hz. Cell extract was kept on ice until use. To assay beta galactosidase activity, 250 μl cell extract was mixed with 100 μl 1 M Tris pH 7.0, 200 μl 4 mg/ml ONPG (Sigma-Aldrich), and 450 μl DI water. The mix was incubated at 37 °C, and readings were taken starting at 30 mins. Activity was calculated as described here^4^, including normalization for protein concentration quantified through Bio-Rad Bradford Assay.

Pyruvate decarboxylase and alcohol dehydrogenase activity was assayed as described in ref. 32 with the following changes. Crude cell extract was prepared as described above for promoter activity assays. Pyruvate decarboxylase activity was measured instead of keto-isovalerate activity. The assay was performed as described using pyruvate instead of alpha-ketoisovalerate as the substrate, and 25 μl cell extract was used. For alcohol dehydrogenase activity, 150 μl cell extract was used and the substrate was acetaldehyde.

### Lactate and ethanol fermentation

The final fermentation media consisted of 500 mM sodium carbonate buffer at pH 9.7, 45 g/l carbon source (D-glucose, D-cellobiose, D-xylose or a mix of cellobiose/xylose), 1mM H_2_PO_4_, 0.1 mM, MgSO_4_, 0.05 mM CaCl_2_, 1 g/l (NH_4_)_2_SO_4_, 200 μg/l (NH_4_)_6_Mo_7_O_24_ · 4H_2_O, 2 mg/l FeCl3, Hutner’s Metals 44 solution (per 1 l 250 mg Na_2_EDTA, 1.095 g ZnSO_4_·7H_2_O, 500 mg FeSO_4_·7H_2_O, 154 mg MnSO_4_·*x*H_2_O, 39.2 CuSO_4_·5H_2_O, 24.8 mg Co(NO_3_)_2_·6H_2_O, 17.7 mg Na_2_B_4_O_7_·10H_2_O) and 10 μg/ml of cobalamin, biotin, and thiamine HCL, each. Modifications in early formulations appear in text. For highest tighter production, all salt and metal concentrations were increased 5-fold, and D-glucose was given at 30%w/v. Fermentation media was prepared with unsterilized tap water in unsterilized glassware, unless otherwise noted in text. Soil was added at 2%w/v. Other water sources were used as noted in text. For sea water, buffer strength was decreased to 250 mM.

Fermentations were performed as follows. A 200 ml overnight culture was grown from a single colony (37 °C, 250 rpm) in ALB with chloramphenicol and 50 g/l of the carbon source to be fermented in a 1 l baffled shake flask. After 16–22 hrs, cells were pelleted by centrifugation at 3500 rcf for 30 min at 16 °C. Cells were suspended in 20 ml fermentation media with 5 μg/ml chloramphenicol. Cells were washed again in 20 ml fermentation media without chloramphenicol, then suspended in 5 ml fermentation without chloramphenicol and placed in 14 ml snap cap tubes. Tubes were press sealed and covered with parafilm. pH was adjusted at 8 and 10 hours with addition of 2–3 drops of 10M NaOH. Error bars represent standard deviation. Carbohydrate consumption as used to calculate percent yield of fermentations is presented in Supplementary Figs 9 (glucose) and 10 (other sugars).

### Analysis of fermentation products

Ethanol was quantified by gas chromatography with flame ionization detection as previously described^16^,^44^. Lactate, succinate, acetate, glucose, cellobiose, and xylose were identified and quantified by HPLC analysis on an Aminex HPX-87H column (Bio-Rad) as described previously, but changing the mobile phase from 5 mM H_2_SO_4_ to 30 mM H_2_SO_4_^44^.

## Additional Information

**How to cite this article**: Wernick, D. G. *et al.* Sustainable biorefining in wastewater by engineered extreme alkaliphile *Bacillus marmarensis*. *Sci. Rep.* **6**, 20224; doi: 10.1038/srep20224 (2016).

## Supplementary Material

Supplementary Information
