# Supplementary material for: Sustainable biorefining in wastewater by engineered extreme alkaliphile Bacillus marmarensis
Source: Sci Rep. 2016 Feb 1;6:20224. doi: 10.1038/srep20224 (PMC4735285; doi:10.1038/srep20224)
Supplement: Supplementary Information [file srep20224-s1.pdf]

Sustainable biorefining in wastewater by engineered extreme alkaliphile *Bacillus marmarensis*

SUPPLEMENTARY INFORMATION

David G. Wernick[1], Sammy P. Pontrelli[1], Alexander W. Pollock[1], James C. Liao[1,2,3,4]*

Affiliations

[1]Department of Chemical and Biomolecular Engineering, University of California, Los Angeles, 5531 Boelter Hall, 420 Westwood Plaza, Los Angeles, CA 90095, USA

[2]Department of Chemistry and Biochemistry, University of California, Los Angeles, 607 Charles E. Young Drive East, Los Angeles, CA 90095, USA

[3]UCLA-DOE Institute for Genomics and Proteomics, University of California, Los Angeles, 201 Boyer Hall, 611 Charles E. Young Drive East, Los Angeles, CA 90095, USA

[4]Department of Bioengineering, University of California, Los Angeles, 420 Westwood Plaza, 5121 Engineering V, Los Angeles, CA 90095, USA

*Corresponding author: James C. Liao, Email: liaoj@seas.ucla.edu, Phone: +1-310-8251656, Fax: +1-310-2064107

Supplementary Figure 1. Plate assay of *B. marmarensis* contamination resistance. Following propagation on purposefully-contaminated, unsterile B. marmarensis cultures, plates were streaked from the mixed cultures and analyzed for growth of contaminats. No major fungal contamation could be discerned. Foreign bacterial colonies observed, but not in great numbers.

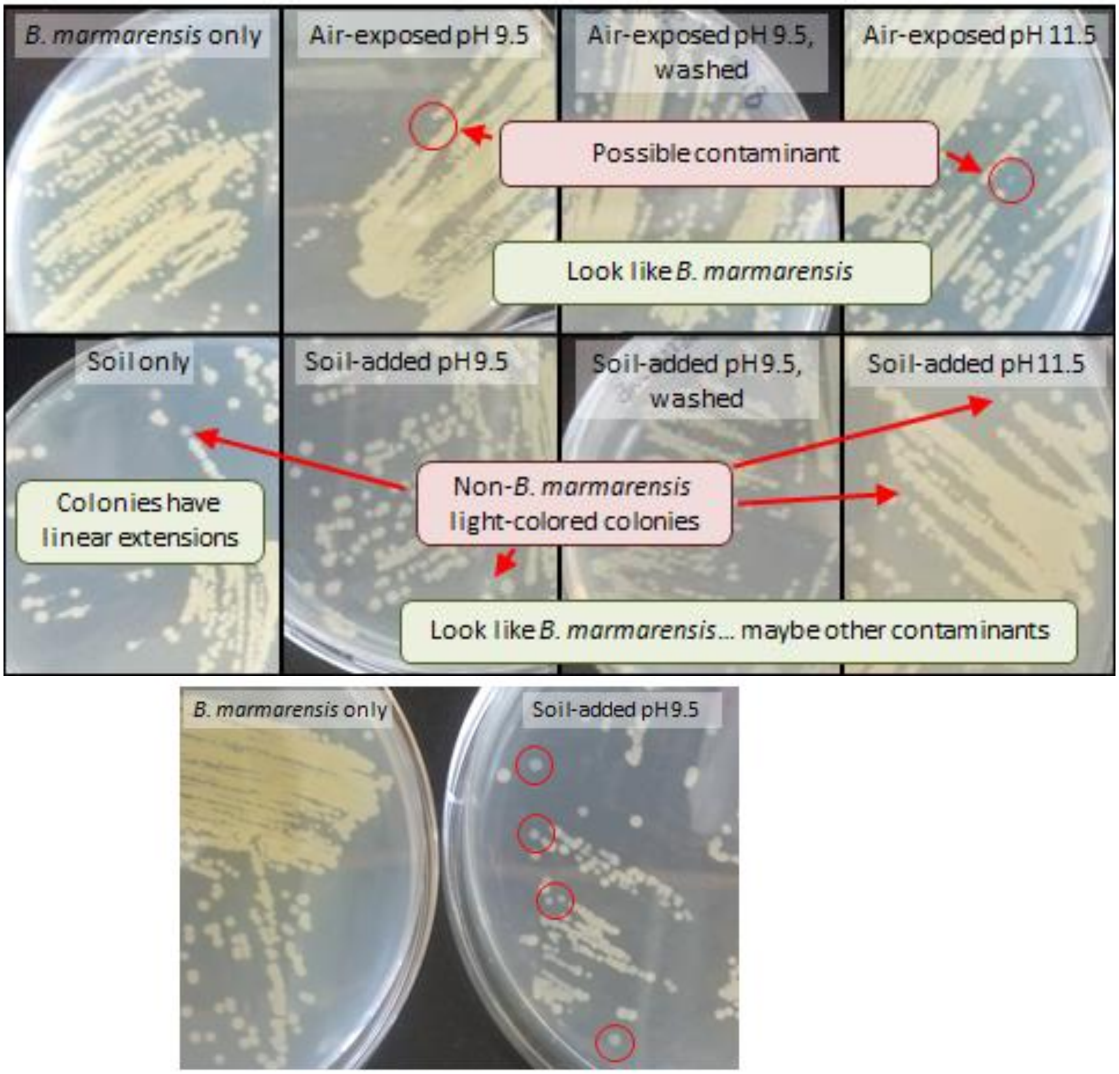

- No major fungal contamination observed.
- Most bacterial colonies in this study look alike, although at least one other species has been identified.
  - Need molecular characterization to study species distributions.

Supplementary Figure 2. High-resolution melt analysis of genomic DNA extractions from contamined-cultures throughout the study. The melt temperature of three hypervariable 16s rDNA regions of each contamined culture was examined over the study. In the open-air contaminated systems, the melt temperatures either directly match throughout or at the end of the experiemt with that found for B. marmarensis monocultures. In soil-contaminated cultures, melt temperatures trend towards that of B. marmarensis overtime, particularly at pH 11.5. B. marmarensis appears to show some dominance of over strains based on its 16s rDNA melting temperatures being matched in this study.

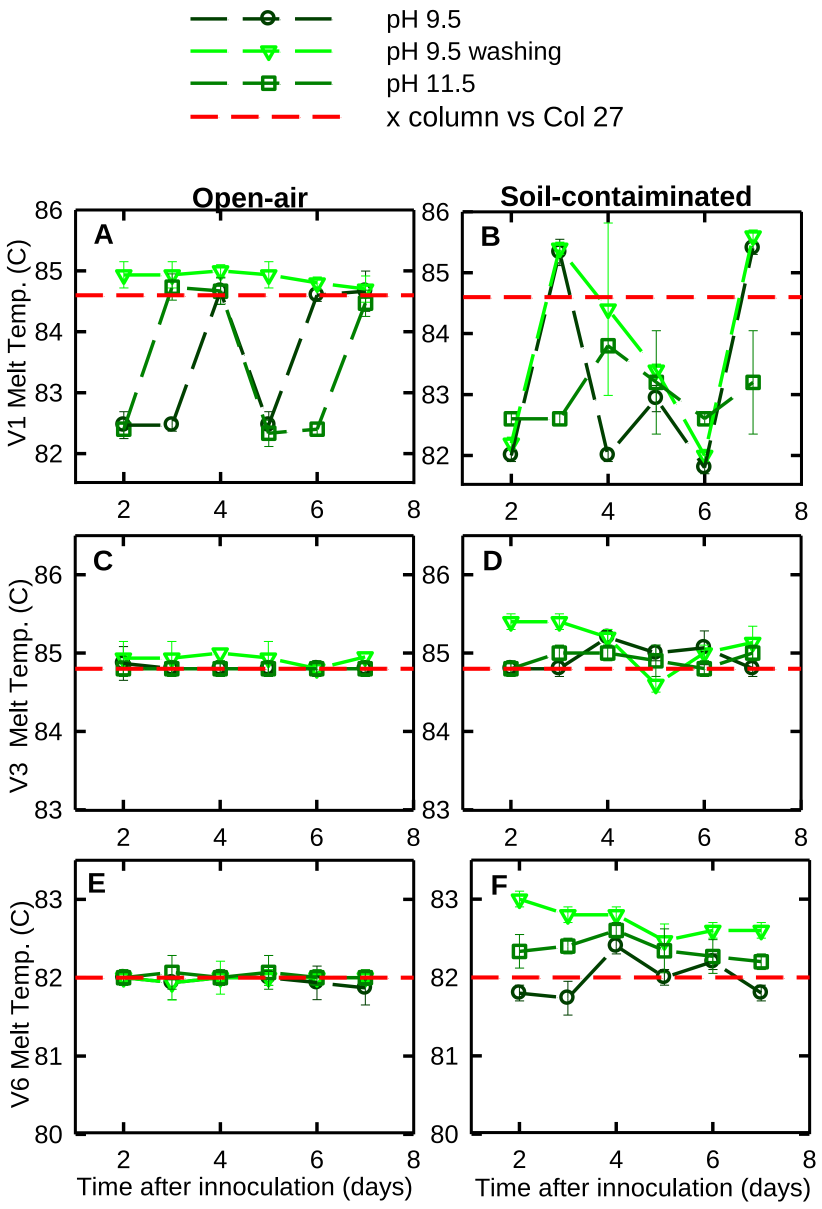

Supplementary Figure 3. Species distribution in 16s rDNA library from the round of contamination studies. 16s rDNA library was built on complete genomic DNA extractions from the final cultures, described further in the main text. The complete distribution of all identified species is given here. *B. sp.* represents strains of the genus *Bacillus* that do not have given species names. Unknown strains are reads that did not return exact matches in BLAST searches.

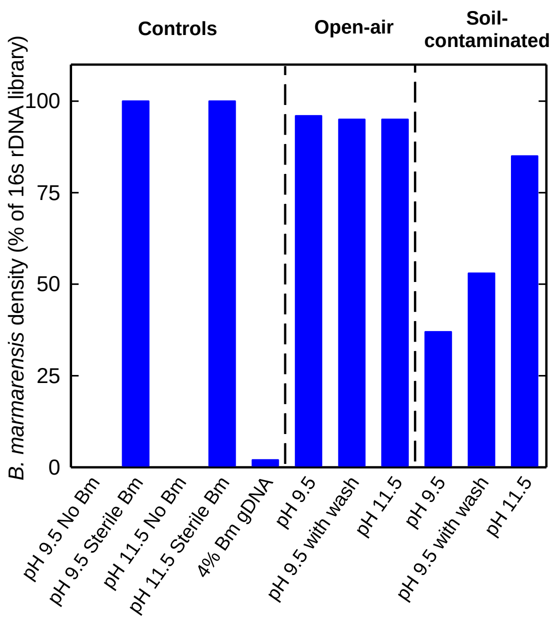

Supplementary Figure 4. Process flow of the eletrotransformation procedure for B. marmarensis. To achieve high-efficiency transformations several adjustments to protocols for other species were made. This included preheating and pre-aerating the diluent media, minimizing glycine treatment time, using fresh wash buffer, applying a square-wave electric pulse, and not using a very-high osmolarity rescue media.

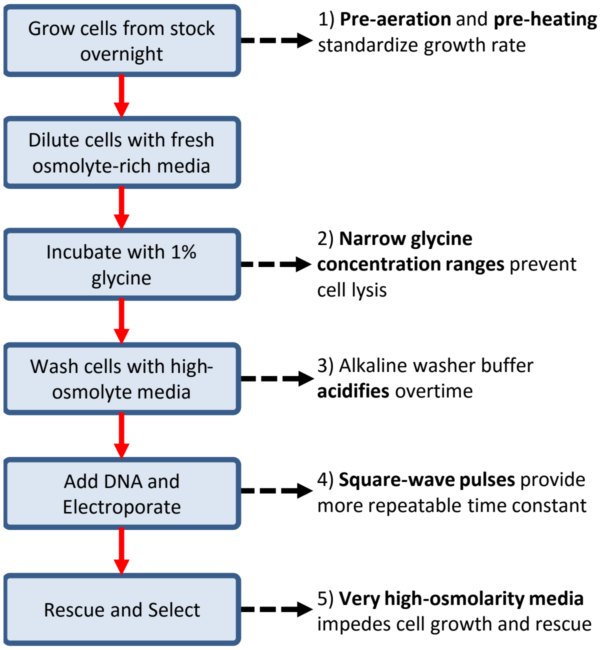

Supplementary Figure 5. Genetic transformation details and data. Eletrotransformation of gram-positive bacteria has been achieved using a general flow sheet as shown in Fig. 5, but previously-unobserved modifications were required for transformation of *B. marmarensis*. The major differences in eletrotransformation of gram-positive strains compared to *E. coli* are the use of sugar alcohols as osmotic stabilizers (eg. D-sorbitol and D-mannitol) and cell-wall weakening agents (eg. glycine or lysozyme) that are not employed for gram-negative strains. However, transformation of *B. marmarensis* required several more modifications. (A) First, following overnight growth liquid media, the strain required pre-heating and pre-aeration of the diluent media for regular growth. Irregular and unpredictable cell growth resulted without both preheating and pre-aerating. (B) *B. marmarensis* showed a small window of sensitivity to glycine pretreatments. In *B. marmarensis*, 1 %(w/v) glycine sufficiently weakened cell walls. However, the glycine addition had to be performed with a cell density (OD_{600nm}) above 0.35 to avoid cell lysis. (C) Only a very narrow range of glycine concentrations was effective. 0.7 %(w/v) glycine did not inhibit cell-wall synthesis, while 1.3% led to significant loss of cell density. This working range of less than 0.6 %(w/v) is significantly smaller than prior transformation protocols. Third, the wash buffer containing glycerol, 0.5 M D-sorbitol, 0.5 M D-mannitol had to be raised to an alkaline pH with minimal addition of base. Excess base would increase the salt concentration and electrical conductivity to lower the overall efficiency of electroporation. (D) As this has not been an issue in previous eletrotransformation protocols, prior research has not shown the acidification of wash buffers. Assembly of fresh wash media bypasses this issue. (E) Although not unique to only *B. marmarensis*, square-wave electric pulses and minimal osmotic stabilizers in the rescue media raised the number of successful transformants. (F) Combining all changes, eletrotransformations reached efficiencies on the order of $1x10^5$ transformants per μg heterologous DNA. (G) and (H) Colony PCRs and plasmid purifications verified successful transformation and maintenance of plasmids. (ON FOLLOWING PAGE).

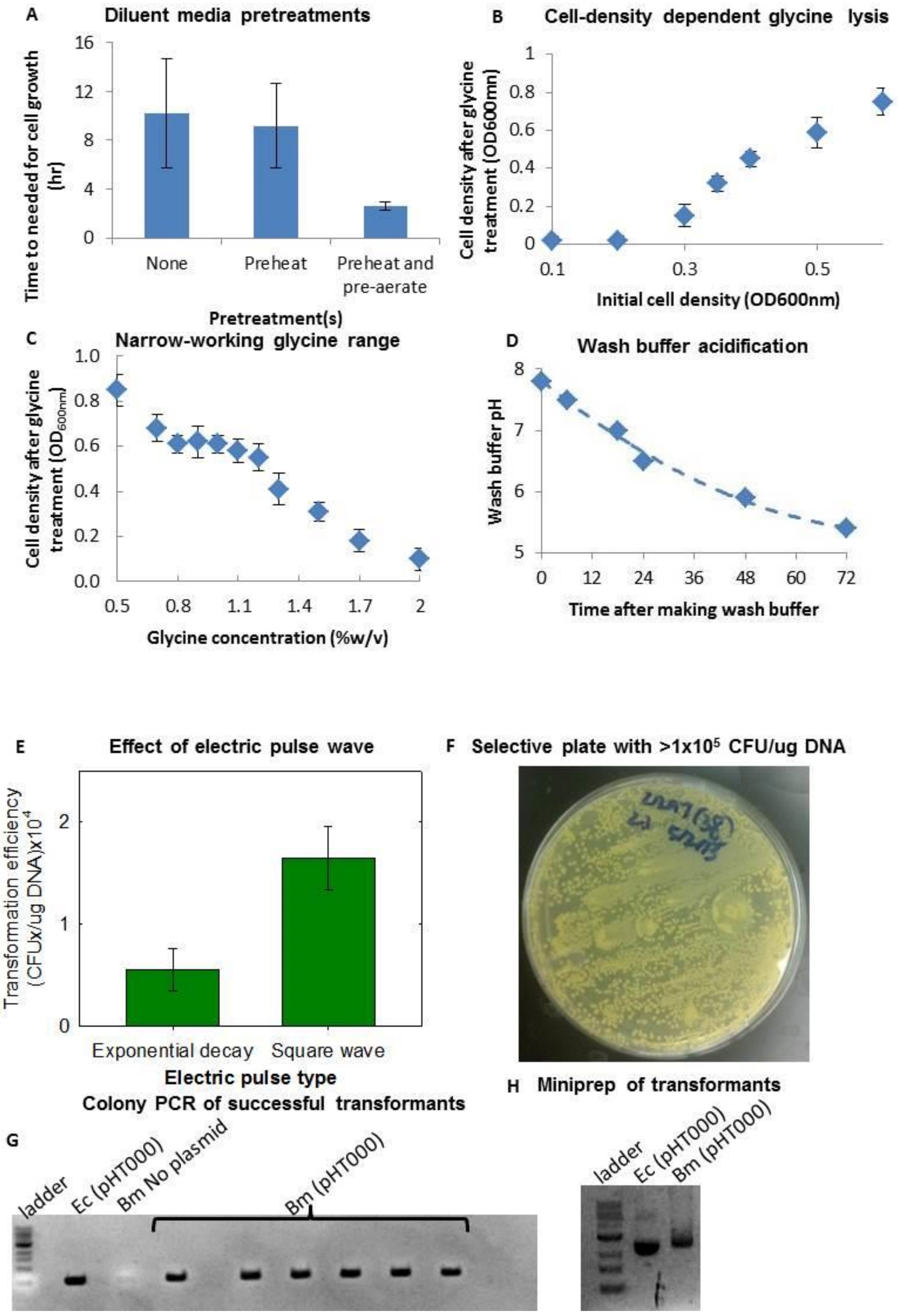

A
Diluent media pretreatments
Time to needed for cell growth (hr)
16
12
8
4
0
None
Preheat
Preheat and pre-aerate
Pretreatment(s)
B
Cell-density dependent glycine lysis
Cell density after glycine treatment (OD600nm)
1
0.8
0.6
0.4
0.2
0
0.1
0.3
0.5
Initial cell density (OD600nm)
C
Narrow-working glycine range
Cell density after glycine treatment (OD600nm)
1.0
0.8
0.6
0.4
0.2
0.0
0.5
0.8
1.1
1.4
1.7
2
Glycine concentration (%w/v)
D
Wash buffer acidification
Wash buffer pH
8
7
6
5
0
12
24
36
48
60
72
Time after making wash buffer
E
Effect of electric pulse wave
Transformation efficiency (CFUx/ug DNA)x10^4
2
1
0
Exponential decay
Square wave
Electric pulse type
F
Selective plate with >1x10^5 CFU/ug DNA
Colony PCR of successful transformants
G
ladder
Ec (pHT000)
Bm No plasmid
Bm (pHT000)
H
Miniprep of transformants
ladder
Ec (pHT000)
Bm (pHT000)

Supplementary Figure 6. Demonstration of plasmid replication and integrity in *B. marmarensis*. (A) Colony PCR confirms the presence of gene transformed into *B. marmarensis* on plasmid pDGW38. (B) Miniprep of plasmids transformed into B. marmarensis shows intact plasmid, although yield was much lower than that from E. coli.

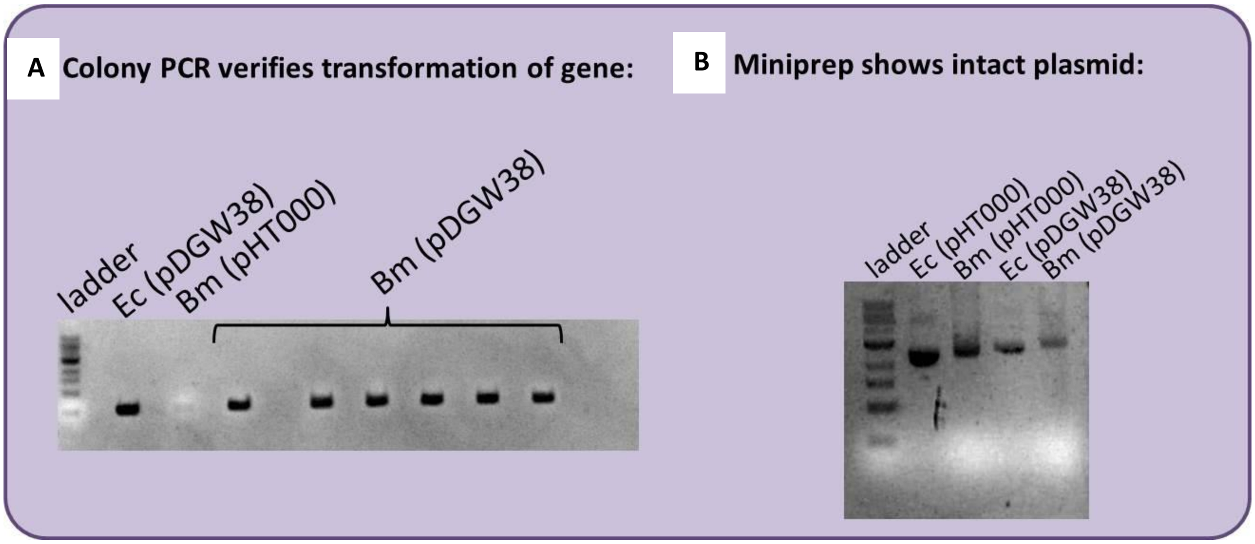

Supplementary Figure 7. Lactate dehydrogenase (LDH) activity in *B. marmarensis* with and without antisense knockdown. (A) Antisense knockdown construct setup. (B) Knockdown of *ldh* activity displayed in strain expressing antisense sequence.

Antisense knockdown setup

A

- **Antisense knockdown in prokaryotes**: Transcribe **reverse-complementary RNA sequence** that will bind mRNA of target gene and prevent translation (block RBS + first few codons).
  16s RNA ACCTCCTTT (aaaggaggt)

- *ldh* promoter, RBSs, and ORF beginning

Promoter

- gaacgtaaataagttgtctgttatgacttgatttagtttctggttttatttaaaatatttatcgagtccttcttggtcatctaa
gttaggtgtcaattcggttacgatttgacaccccatatccaagaaggctcctttttacatttatgtttgtgaaatgtttcac
aagttagacgtaactatttatatgaagtggtagattttagacatttttaacatagactaaggggagttaagggatattt
acgaggaggtatgacaATGAAAGTTTCATTATTT...

RBS 1 RBS 2
RBS 3 ORF

- Antisense construct:

Promoter

- gaacgtaaataagttgtctgttatgacttgatttagtttctggttttatttaaaatatttatcgagtccttcttggtcatctaa
gttaggtgtcaattcggttacgatttgacaccccatatccaagaaggctcctttttacatttatgtttgtgaaatgtttcac
aagttagacgtaactatttatatgaagtggtagattttagacatttttaacatagactaaggggagttaagggatattt
acgaggaggtatgacaATGAAAGTTTCATTATTT...

Reverse complement of Antisense RNA

B

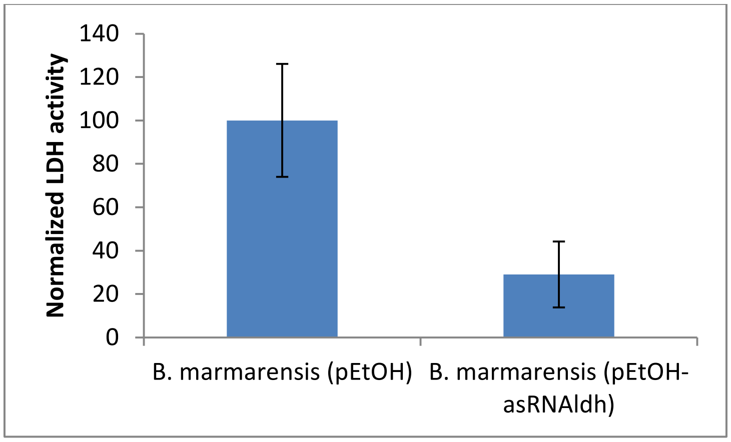

Supplementary Figure 8. *B. marmarensis* cell mass was re-cycled for additional ethanol production. Yields showed slight drops in successive batches, but overall produced a cumulative titer of 37 g/l at 55% the theoretical maximum.

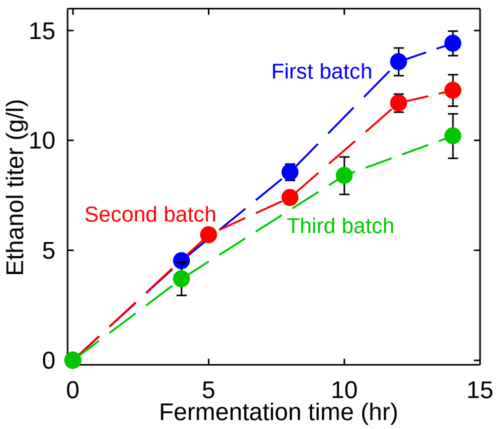

Supplementary Figure. 9: Supporting fermentation data for ethanol yield calculations from glucose. Glucose consumption through fermentation of B. marmarensis harboring plasmids (A) pEtOH, (B) pEtOH-asRNA, (C) pEtOH-asRNA156. (D) and (E) Fermentations with *B. marmarensis* (pEtOH-asRNA156) with extra glucose and salts. (F) Pyruvate decarboxylase (PDC) and alcohol dehydrogenase (ADH) activity assays in *B. marmarensis* WT, (pEtOH), and (pETOH-asRNA).

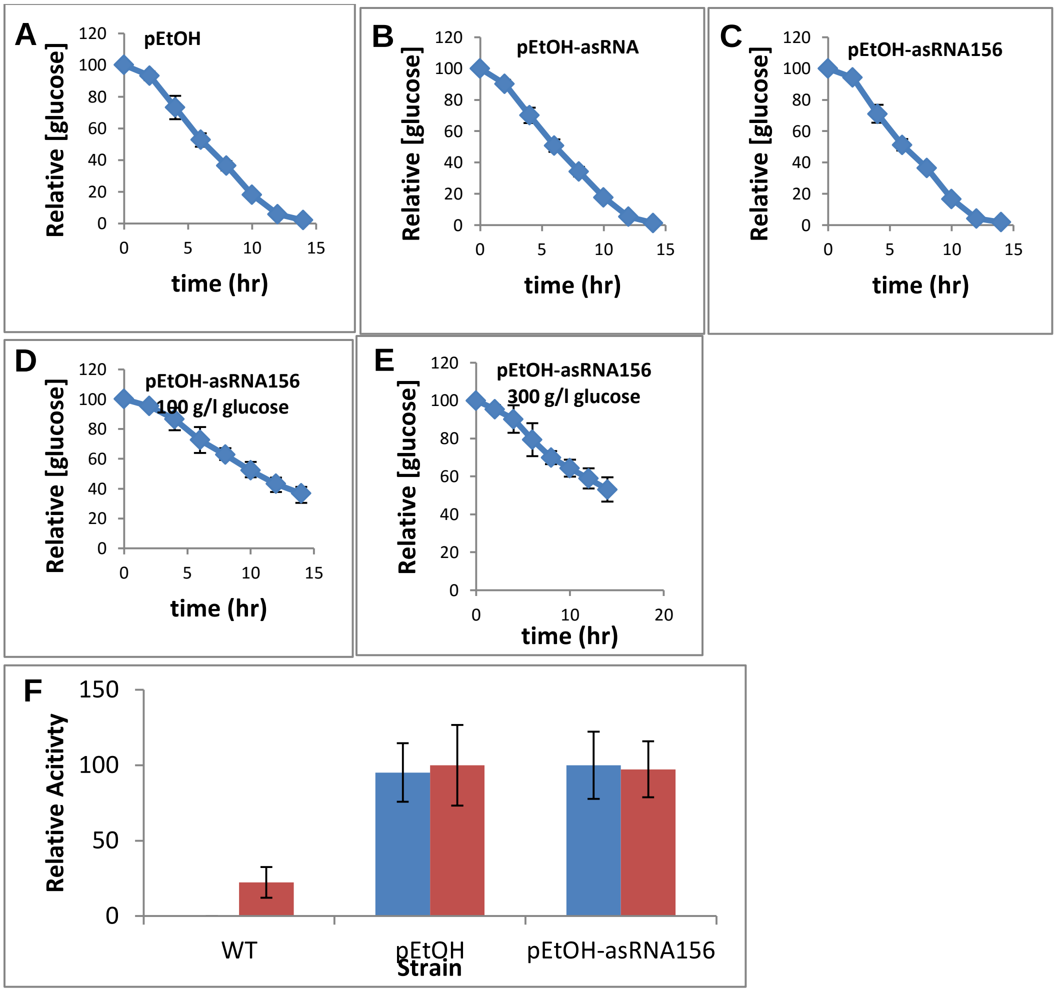

Supplementary Figure 10: Supporting fermentation data for ethanol for yield calculations from cellobiose and xylose. Carbohydrate consumption in fermentations of (A) cellobiose, (B) xylose, and (C) cellobiose and xylose. (D) Ethanol titers from cellobiose, xylose, and cellobiose/xylose mixtures with contamined conditions.

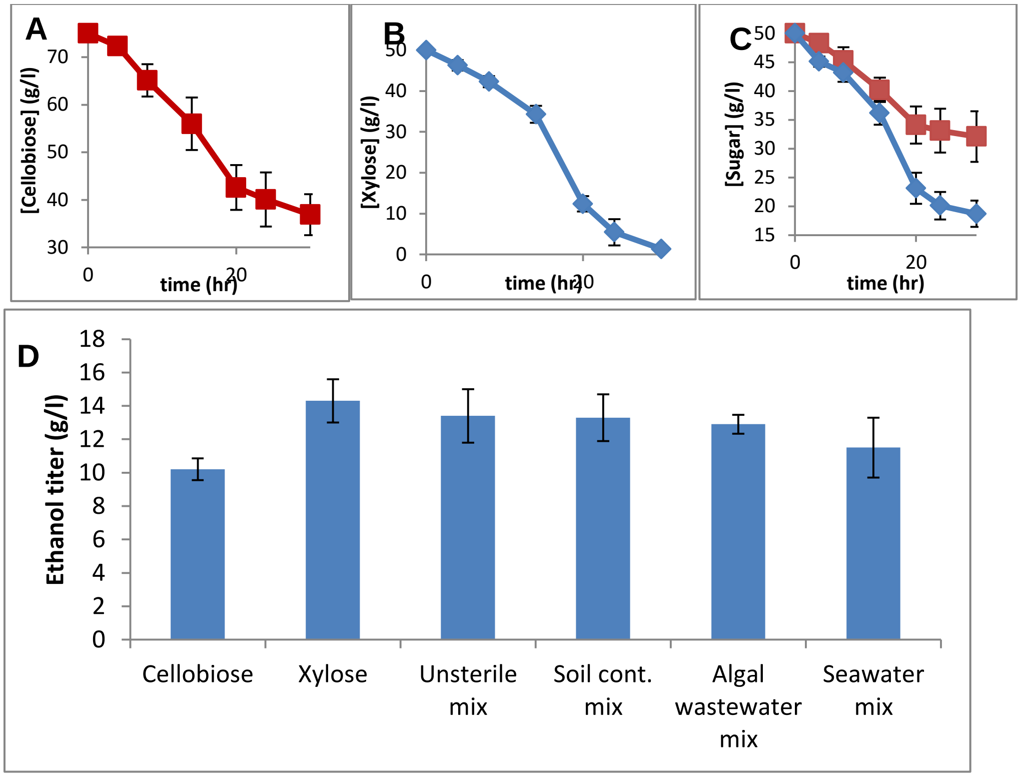

Supplementary Table 1. Primers and genes for *B. marmarensis* promoter library

RAST#	Gene	Forward/reverse primer
14	CdpS	cagctatgaccatgattacgcctaagtaaactgcatatcctgttacgag
		TTAGGAAATTGGATAATAGTCATCTC ATG ACC TCC CTT CAG GTG A
23	CTPs	cagctatgaccatgattacgccAGA GGA CGA GCT AGA TGA AAT CTC
		TTAGGAAATTGGATAATAGTCATGCG ATC GAC TCC TCT ACT TTT CAT C
31	Rrna	cagctatgaccatgattacgccCAG TTA GAC AAG CTA TGG GCG ATC C
		TTAGGAAATTGGATAATAGTCATTGA TTT CAT CTC CTT CCG CCC TGA ATC
44	R5P Iso	cagctatgaccatgattacgccttacagcaatggcagagcaaca
		TTAGGAAATTGGATAATAGTCATctgtacaacctcctaaatggttttaagaaaag
50	atpI	cagctatgaccatgattacgcccaacgcgattgtaagagcaacc
		TTAGGAAATTGGATAATAGTCATgaacaccgctcccctcaag
59	atpE	cagctatgaccatgattacgccTGCATCAACATCTCGTGCACTT
		TTAGGAAATTGGATAATAGTCATGTGAGAAACCCTCCTCGAGTAGC
123	aap	cagctatgaccatgattacgccTCCAATGGTTTGGGTTTGAGAA
		TTAGGAAATTGGATAATAGTCATCCACACCCTTCCTTCTTCTGATTC
152	DHPs	cagctatgaccatgattacgccCGCGCGTTAACACTAAGCAGAT
		TTAGGAAATTGGATAATAGTCATCTTATACCACCCCCAACCAGTAATAG
440	murE	cagctatgaccatgattacgccAAAGTCGATGCGACAGGAGAAG
		TTAGGAAATTGGATAATAGTCATGTTTAACCCTCACATTCAAACCTTTTTC
442	mraY	cagctatgaccatgattacgccAAGCTTACTGGCAGCAAAGGTG
		TTAGGAAATTGGATAATAGTCATTGCTAATCATTCCTCCTTTCTTTTTG
443	murD	cagctatgaccatgattacgccTTTTGGTGCGTTTGCTATTTTG
		TTAGGAAATTGGATAATAGTCATGTATGTTTACACCTCATTTACATCCATACC
569	comk	cagctatgaccatgattacgcccggatttccagtatggcttgtg
		TTAGGAAATTGGATAATAGTCATagggaatcgctccttagtgga
577	S-layer	cagctatgaccatgattacgccTCGTCGATTTTTGACGAAATTG
		TTAGGAAATTGGATAATAGTCATAAGTATAATTCCTCCTTCAAATTTGC
588	5Nuc	cagctatgaccatgattacgccTGAGCAAACTGCCAAGCAGTAA
		TTAGGAAATTGGATAATAGTCATCATATTCCTCCTAGAAATCTATTTTCCACTTAG
595	s-layer	cagctatgaccatgattacgccACCCATGATTGCCAGCTTATGA
		TTAGGAAATTGGATAATAGTCATGATCATTCCTCCTACAAAATAGTCACATTC
891	sigB	cagctatgaccatgattacgccaattgccaatcgccttggat
		TTAGGAAATTGGATAATAGTCATttttctccacctcatctccgcga
1045	GDH	cagctatgaccatgattacgccGCCTGATTCATCCGCTCTAA
		TTAGGAAATTGGATAATAGTCATTTCAATCTCCCCCAATATCG
1361	s-layer	cagctatgaccatgattacgccCTTGGTTCGGGTGACGATAC
		TTAGGAAATTGGATAATAGTCATAAGATAACCTCCAATATGGTAGAAAATAGT
1486	BCAAtr	cagctatgaccatgattacgccGCTTGATGAAGCAGCAAAGGTT
		TTAGGAAATTGGATAATAGTCATCACTACTCCTCCTCAACATAATCTACG
1502	tns-cdd	cagctatgaccatgattacgcccgcggatttactcgcctgtt
		TTAGGAAATTGGATAATAGTCATttaaagaggatctcctctcctgattgc
1503	cdd	cagctatgaccatgattacgccgggagaagtccagcgggtaa
		TTAGGAAATTGGATAATAGTCATtgttctcgtcccttctaatggtcc
1513	rpoD	cagctatgaccatgattacgccgcatcatgccattgcagctt
		TTAGGAAATTGGATAATAGTCATtcgatcccctccttccattgt
2070	Alk	cagctatgaccatgattacgccCAT AGG CAT TTG TCT GCA AAG GCC

		TTAGGAAATTGGATAATAGTCATGAT ACA TAC ATA AGG AGG GTG AGA CCT A
2126	GDH	cagctatgaccatgattacgccATATGCACCGGATGGAATGAAC
		TTAGGAAATTGGATAATAGTCATATTATCCCCTCCCAAAATCTGC
2168	GS	cagctatgaccatgattacgccGCGAGAAGCTGTTGCTAGTG
		TTAGGAAATTGGATAATAGTCATCATCCACTTCCTTTTCATAAAGTAT
2196	GaPAT	cagctatgaccatgattacgccAACGGCTGTTCTAAGCGAGTACG
		TTAGGAAATTGGATAATAGTCATGGTTTCCCCCCAAGTGTTCC
2804	GDH	cagctatgaccatgattacgccCACCTCGTGGAAAAACTCGT
		TTAGGAAATTGGATAATAGTCATCTTCTCACCCCGCCAATA
2910	GS	cagctatgaccatgattacgccAATGATGGCAGGAAGAAACG
		TTAGGAAATTGGATAATAGTCATTGTTATGTATTCCCCTTTCAAATTCT
3351	sig70	cagctatgaccatgattacgccttactggggcaggcaacgta
		TTAGGAAATTGGATAATAGTCATtgcgactacccccgaagttg
3358	GDH	cagctatgaccatgattacgccATGACGCCAACAAGCCTCTAT
		TTAGGAAATTGGATAATAGTCATCTGTTTCACCTCTAGCCAGTGTTC
3793	IlvE	cagctatgaccatgattacgccCGGCAGCTATTCTCGTTTTC
		TTAGGAAATTGGATAATAGTCATGTTGCCAAGCTCGTTTTACA
3856	malDH	cagctatgaccatgattacgccTGCACAAGTTGGCGGTATTG
		TTAGGAAATTGGATAATAGTCATCTTCATCAACTCCCAGTTATGATAGTGA
3858	cit syn	cagctatgaccatgattacgccATGCGTGGATTGCATTAGCC
		TTAGGAAATTGGATAATAGTCATGTTAATCTCTCCTTTTCCCTAATTATCTTTTT

Supplementary Table 2. Primers to clone pHTetOH series plasmids

pHT backbone	AGCAGCATCCGGATAGAGGCTTGTTGGCGTCATttgatatgcctcctaaatttttatcta
pHT backbone	TAACAAGCTCCTCTAGTAAGGAGGAACTACTATGAACTTTAATAAAATTGATTTAGACAA
P3358	tttagataaaaatttaggaggcatatcaaATGACGCCAACAAGCCTCTAT
P3358	GAAAGGAATATAAAAAGTTGAAGAAGCCATCTGTTTCACCTCTAGCCAGTGT
adhA (Zm)	ATACATCGAACACTGGCTAGAGGTGAAACAGATGGCTTCTTCAACTTTTTATATTCCTTT
adhA (Zm)	TACATTTTTTGTATCGTCACCCGAACCAAGTTAGAAAGCGCTCAGGAAGAGT
P1361	GTTGAAGAACTCTTCCTGAGCGCTTTCTAACTTGGTTCGGGTGACGATACA
P1361	GCTAAATAGGTACCGACAGTATAACTCATAAGATAACCTCCAATATGGTAGAAAATAG
pdc (Zm)	ACTATTTTCTACCATATTGGAGGTTATCTTATGAGTTATACTGTCGGTACCTATTT
pdc (Zm)	aaatcaattttattaaagttcatagtagttcctccttaCTAGAGGAGCTTGTTAACAGGC
phtET2 amplify	cccTAAAAGTAATTACATTAATGACGCCAACAAGCCTCTA
phtET2 amplify	aatcaagtcataacagacaacttatttacgttgatatgcctcctaaatttttatcta
Pldh w/o rbs	aaatttaggaggcatatcaacgtaaataagttgtctgttatgact
Pldh w/o rbs	AAATCACGGAAAAGCttagtctatgttaaaaaatgtctaaaatct
asRNA	catttttaacatagactaaGCTTTTCCGTGATTTAGATCG
asRNA	GGCGTCATTAATGTAATTACTTTTAgggggagttaagggatatttac
pHtetOH amplify	ggatatttacgaggaggtatgacAATGAGTTATACTGTCGGTACCTATTT
pHtetOH amplify	ggagctctaactcgctaacaacctcTTAGAAAGCGCTCAGGAAG
Pldh	AGAACTCTTCCTGAGCGCTTTCTAAgaggttgttagcgagttagag
Pldh	AATAGGTACCGACAGTATAACTCATTgtcatacctcctcgtaaatatc
pHTetOH45/98/156 part1 rev	gaaagaacatgtgagcaaaaggcca
pHTetOH45/98/156 part2 fwd	tggccttttgctcacatgttctttc
pHTetOH45 part1 fwd	ACCATAAAAAAGAAAACAAAGAAGGAGGGTAAATATGAGTTATACTGTCGGTACCTATTT
pHTetOH45 part2 rev	CCTCCTTCTTTGTTTTCTTTTTTATGGTAGAAAATAGTTTAATAGATAGATATTACTAGT
pHTetOH98 part1 fwd	ATACGTAAGTAAGAAAACACGAAGGAGGGAAGTTATGAGTTATACTGTCGGTACCTATTT
pHTetOH98 part2 rev	ACTAGTAATATCTATCTATTAAACTATTTTCTACCATACGTAAGTAAGAAAACACGAAGG
pHTetOH156 part1 fwd	TAAATTTTAATAACACAGAAGAAGGAGGTAGAAAATGAGTTATACTGTCGGTACCTATTT
pHTetOH156 part2 rev	ACTAGTAATATCTATCTATTAAACTATTTTCTACCATAAATTTTAATAACACAGAAGAAG
